# Our Contemporaries

**Published:** 1918-08

**Authors:** 


### OUR CONTEMPORARIES

---

**Contents and Index of the Philippine Journal of Science,** Volume I (1916) to Volume X (1915). Manila. This beautiful index, of 442 pages, is in keeping with the Journal to which it forms a complete guide. A glance at its pages gives one a feeling of pride in the amount and character of scientific work accomplished by our fellow citizens in the Philippines.

